# Waiting times before dental care under general anesthesia in children with special needs in the Children's Hospital of Casablanca

**DOI:** 10.11604/pamj.2014.17.298.2714

**Published:** 2014-04-20

**Authors:** Bouchra Badre, Zineb Serhier, Samira El Arabi

**Affiliations:** 1 Pediatric dentistry unit, faculty of dentistry of Casablanca; 2 Medical informatics laboratory, Faculty of Medicine and Pharmacy of Casablanca

**Keywords:** Child with special needs, dental care, general anesthesia

## Abstract

**Introduction:**

Oral diseases may have an impact on quality of children's life. The presence of severe disability requires the use of care under general anesthesia (GA). However, because of the limited number of qualified health personnel, waiting time before intervention can be long. Aim: To evaluate the waiting time before dental care under general anesthesia for children with special needs in Morocco.

**Methods:**

A retrospective cohort study was carried out in pediatric dentistry unit of the University Hospital of Casablanca. Data were collected from records of patients seen for the first time between 2006 and 2011. The waiting time was defined as the time between the date of the first consultation and intervention date.

**Results:**

127 children received dental care under general anesthesia, 57.5% were male and the average age was 9.2 (SD = 3.4). Decay was the most frequent reason for consultation (48%), followed by pain (32%). The average waiting time was 7.6 months (SD = 4.2 months). The average number of acts performed per patient was 13.5.

**Conclusion:**

Waiting times were long, it is necessary to take measures to reduce delays and improve access to oral health care for this special population.

## Introduction

Dental cares are major unmet health needs of persons with special needs. Studies have shown that these people suffer from oral disease more than the general population [1–4]. Intervention difficulties are an aggravating factor. In fact, dental care is more difficult to achieve in the presence of cognitive impairment or behavioral disorder [5, 6]. Difficulties in cooperation undermine the process of care in the dental chair and are a major cause of treatment failure. The presence of severe disability is sometimes a contraindication of care in dental chair [7]. Hence, the use of health care under general anesthesia (GA) represents an alternative choice to ensure complete cleaning up of the oral cavity in one session in children with special needs [5, 7].

However, because of the limited number of qualified health personnel waiting times before dental care under GA are long. In a Quebec study, these periods ranged from 6 to 12 months [8]. During this waiting period, the oral health status of the child may worsen and impact his overall health and quality of life. The evolution of untreated dental disease can lead to acute or chronic painful episodes of varying intensity that is not spontaneously reported because of the difficulty of expression of these persons [5, 9, 10].

In Casablanca, the collaboration between pediatric dentistry unit and pediatric surgery department of the Children′s Hospital of IBN RUSHD helped support children with special needs for dental treatment under GA. The existence of two public structures in Morocco for dental care under GA, affects the waiting time. Indeed, this support is not made in other structures because it requires special skills that are not part of the initial training course of dentists.

The main objective of this work was to evaluate the waiting period before dental care under GA for children with special needs in Morocco. Secondary objectives were to describe the reasons for consultation and the procedures performed.

## Methods

This is a retrospective cohort study carried out in the pediatric dentistry unit of Children′s Hospital Abderrahim Harrouchi of Ibn Rushd University Hospital in Casablanca.

Data was collected from patients’ records that have benefited from dental care under GA. Patients examined for the first time between January 2006 and December 2011 were selected.

The variables studied were socio-demographic including age, sex, and type of payment; (mutualist, paying, and indigent), the type of disability, the reason for consultation, the waiting period, the nature care and duration of act. Indigent patients are patients of low socio-economic level, who receive free care. The waiting time was defined as the time between the date of the first consultation and surgery date. The data processing was done by the SPSS 16.0 software.

## Results

**Figure 1:**
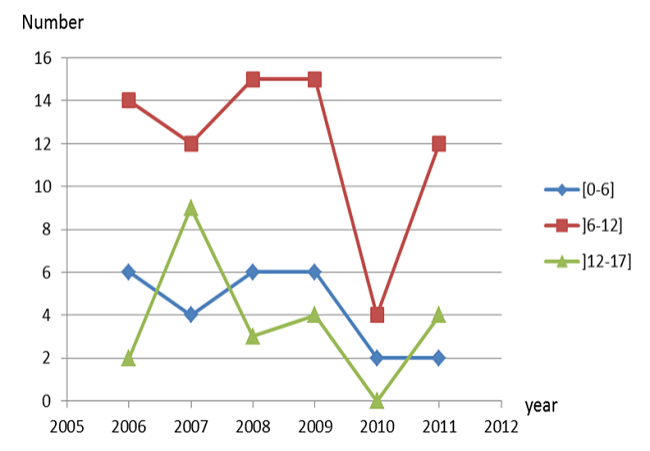
Number of patients by year and age

During the study period, the number of patients who received dental treatment under GA were 127 patients with an annual average of 21.16 patients. The number of children treated under GA ranged from 8 in 2010 to 26 patients in 2009. Among these patients, 73 were male (57.5%), the average age was 9.2 with a standard deviation of 3.4. The most represented age group was that of 6 to 12 (Figure 1). Most of the patients were from Casablanca (82.7%) and 6.3% from El Jadida located 100 km from Casablanca. The rest (11%) came from other regions of Morocco. The majority of the patients treated were indigent (68%) and 14% mutualist. The cerebral palsy was the most common disability (54%). The majority of patients (109) consulted by their own initiative or was referred by their treating physician and 18 patients (14%) were referred by the pediatric dentistry unit due to a failure in nitrous oxide sedation.

**Figure 2:**
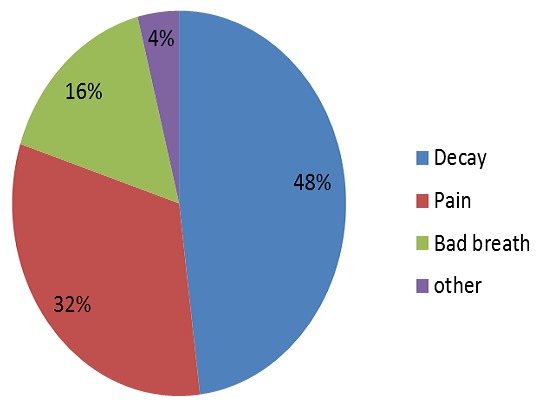
Distribution of patients according to the pattern of consultation

The most frequent reasons for consultation were decay (48%), followed by pain (32%) (Figure 2). The average waiting time was 7.6 months with a standard deviationof 4.2 months; these delays did not differ by year of treatment (Table 1). The average act duration was 92.4 minutes per patient with (SD= 30.0 min).

**Table 1:** Waiting time before care under general anesthesia by year

Year	Waiting time Average (standard deviation)	p
**2006**	6.05 (4.59)	0.174
**2007**	8.24 (3.34)	
**2008**	8.81 (3.98)	
**2009**	7.00 (3.68)	
**2010**	8.75 (4.43)	
**2011**	6.90 (4.80)	

**Figure 3:**
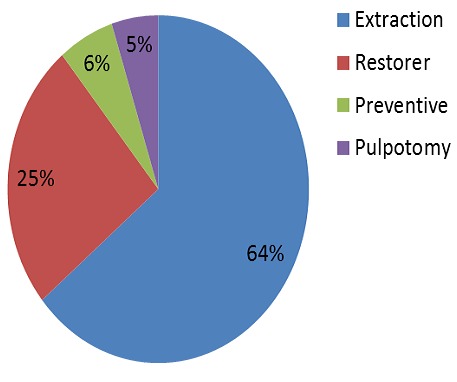
Proportion of procedures performed in primary dentition according to their nature

**Figure 4:**
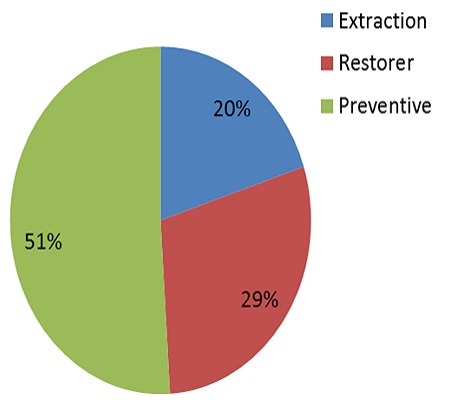
Proportion of acts performed in the permanent dentition according to their nature

The total acts performed were 1719, with an average of 13.5 acts per patient (7.2 acts per patient on deciduous teeth and 6.3 acts per patient on permanent teeth). Approximately 44% of the interventions performed were extractions, these were more frequent in primary dentition (Figure 3, Figure 4).

## Discussion

The average waiting time was 7.6 months with a standard deviation of 4.2 months. Depending on the year, there was no difference between these periods. This average is higher than that observed in France [11] (1.3 to 3.3 months).

It has been reported in the literature that a long waiting time may impact on the health of these children. Indeed, Wogelius et al showed that delays in care could lead to a deterioration of the children's teeth, therefore, a delay of more than four months should be considered as relatively long [12]. Another study showed that during the waiting period, 41% of parents stated that their children needed analgesics, 28.5% stated sleep disturbance of children, 32.9% showed chewing problems and 49.4% of children took antibiotics [13]. The number of patients who received dental care under GA during the study period is low 21.16 / year compared with the number stated in other studies that was 54 to 60 patients year [14, 15].

The small number of children in care under GA in our structure is due to the weekly schedule of patients, and the mutualization of the surgical unit causing cancellations due to urgent interventions. The lowest number of patients treated was noted in 2010 due to the renovation of the operating room during that year.

The majority of patients (82.7%) were from Casablanca, given the nearness and easy access to the health centre, the concept of local support was also highlighted in a study carried out in 1996 in Rennes [11] and another conducted in the UK in 1997 [16]. Unlike developed countries, which have several centres for these patients, Moroccan children with special needs can benefit from dental treatment under general anesthesia at the level of the two centres of Casablanca and Rabat. The absence of other public facilities in the rest of the country shows that some of these children with special needs do not have access to dental care.

Most of the patients had a low socio-economic level, hence the need to improve accessto care in the public sector with the creation of other regional centres. The average age of the patients was 9.2 ± 3.4 with the most age represented group between 6 and 12 (58.3%). This result is consistent with those stated in the literature with the average age ranging from 6.5 to 9.5 [11, 15, 17, 18].

Many studies show that the average age of children with specific needs supported in AG was higher than that of healthy patients [17, 19–22]. This age difference can be explained, according to Haubek et al [17] by the fact that children have special needs in their daily lives, health needs of more severe and complicated than dental problems, which could delay their use of dental care. On the other hand, people with disabilities hardly express their care needs; consultations are often decided by the family upon subjective elements [5]. Dental decay was the main reason for consultation (48%), followed by pain (32%). In other studies, dental decay is usually the first reason for consultation [23, 24].

On the whole, dental extractions were the most performed acts 44%, this result is similar to that found in other studies [21, 22]. The high number of extractions can be explained by the late consultation of these patients. The percentage of extractions in the permanent dentition remains high (20%) despite the predominance of conservative acts. According to Peretz et al [15], permanent teeth extractions were more frequently performed in patients with special needs, which may indicate that dentists prefer to extract severely questionable decayed permanent teeth for this group of subjects rather than risking the likely need for reprocessing.

The achievement of care under GA is still underdeveloped and cannot meet all requests. To reduce the waiting period, responsible have to be aware of the need to create specialized centres for performing acts under GA. Training of qualified staff to provide such care to compensate for the disadvantage in oral health of children with cognitive or mental impairment. The particular practice of this specialty is little or not yet sufficiently taught in the training. It is part of the curriculum of residency in pediatric dentistry; it requires postgraduate training in university hospital for other practitioners.

It is also important to strengthen prevention and education for oral health in this population. The medical, paramedical and educational staffin contact with these children should emphasize the importance of brushing teeth, low sweet diets [17] and regular visits to the dentist.

Appropriate dental care to patients with special needs improves the patients’ quality of life, removing pain and bad breath [22].

## Conclusion

This study shows that the waiting time of children with special needs before specific dental care under GA is long. It is necessary to take measures to improve access to oral health care for this special population.
